# Breath Figure Method for Construction of Honeycomb Films

**DOI:** 10.3390/membranes5030399

**Published:** 2015-08-28

**Authors:** Yingying Dou, Mingliang Jin, Guofu Zhou, Lingling Shui

**Affiliations:** Institute of Electronic Paper Displays, South China Academy of Advanced Optoelectronics, South China Normal University, Guangzhou 510006, China; E-Mails: douyingying@scnu.edu.cn (Y.D.); jinml@scnu.edu.cn (M.J.); zhougf@scnu.edu.cn (G.Z.)

**Keywords:** honeycomb film, breath figure method, organic-inorganic hybrid, self-assembly, nanoparticles, polyoxometalates, quantum dots

## Abstract

Honeycomb films with various building units, showing potential applications in biological, medical, physicochemical, photoelectric, and many other areas, could be prepared by the breath figure method. The ordered hexagonal structures formed by the breath figure process are related to the building units, solvents, substrates, temperature, humidity, air flow, and other factors. Therefore, by adjusting these factors, the honeycomb structures could be tuned properly. In this review, we summarized the development of the breath figure method of fabricating honeycomb films and the factors of adjusting honeycomb structures. The organic-inorganic hybrid was taken as the example building unit to discuss the preparation, mechanism, properties, and applications of the honeycomb films.

## 1. Introduction

Membranes play an important role in science and technology, such as the applications of ion exchange membranes in separation [1,2,3], wastewater treatment [2,3,4], food, and bio-technology [3,4]; nanoporous membranes in blood purification [5], separation and gas removal [6,7], virus filtration [8] and DNA/gene detection [9,10]; liquid crystal polymer membranes in optics, electronics, display devices, and bio-technology [11,12,13,14]; honeycomb films in anti-bacterial activity [15,16], cell/bacteria culture [17,18], protein adsorption [19], tissue engineering [20], lab-on-fiber technology [21], electrowetting display [22], and water/oil separation [23].

To prepare these functional membranes, self-assembly has been widely employed, such as in the examples of the solution casting technology for the preparation of the ion exchange membrane [24] and the breath figure process for honeycomb film formation [16]. Self-assembly is a “bottom-up” method [25], which can spontaneously induce the formation of diverse ordered structures, such as vesicles, nanoporous membranes, nanotubes, and nanofibers, *etc.* The driving forces are mainly the weak interactions such as electrostatic interaction, hydrogen-bond interaction, van der Waals force, capillarity, steric effect, dipolar force, and hydrophobic interaction. Generally, several weak interactions work together to complete one self-assembly process, although one plays the key role. For example, in an ionic self-assembly process, the electrostatic interaction plays the main role; however, the steric effect becomes significant when the molecule is large, and the hydrogen-bond interaction has to be considered for an active hydrogen system. Various materials could be applied for this process, such as surfactants [26], polymers [27,28], nanoparticles, and the organic-inorganic hybrid materials [29,30,31,32,33].

Breath figure patterning [34,35,36,37] is an example of a self-assembly process which forms honeycomb-structured films with micro-pores arranged in honeycomb morphology. The process typically includes several sections: one drop of polymer solution is casted on a substrate surface with humid air flowing up the polymer solution drop; the air/solution interface temperature reduces below the dew point as the solvent evaporates; water droplets condense on solution stabilized by the solutes without coalescence; water droplets align into ordered arrays due to the capillary and Marangoni forces; solutes precipitate around water droplets as solvent continues evaporating; and ordered polymer pore film is formed after the solvent and water droplets evaporate completely [34,35]. There are several key components during the breath figure process: the solute, *i.e.*, the building unit, the solvent, and the humid air. Among them, the building units play an important role which brings the main functions for the honeycomb films. The commonly used building units include amphiphilic copolymers, star polymers, block copolymers, polymeric polyion materials, and organic-inorganic hybrids [34,35]. These materials can dissolve in organic solvents such as carbon disulfide, chloroform, and toluene. The organic-inorganic hybrid is a representative building unit which combines characteristics of both organic and inorganic materials, and therefore obtains multi-functional properties [34,35,36,37,38].

In this review, we focus on the fabrication of honeycomb films using the breath figure method. The mechanisms and developing history of the breath figure method are introduced in the first section, in which the driving forces are also discussed. The second section focuses on the materials and affecting factors for honeycomb film formation using the breath figure method. The materials, formation mechanism, and applications of the organic-inorganic hybrid honeycomb films are discussed in the last section.

## 2. Mechanism of Honeycomb Film Formation: Breath Figure Method

### 2.1. Development of the Breath Figure Method to Form Honeycomb Films

**Figure 1:**
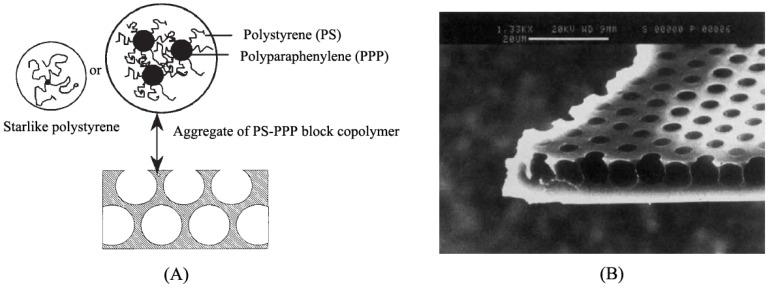
(**A**) Schematic drawing of the honeycomb film formation of star PS polymer or PS-PPP block copolymer; (**B**) SEM photograph of the PS-PPP honeycomb films, with copyright permission from [42].

In 1893 and 1911, the breath figure process was proposed as a water droplet condensation procedure on a solid surface by Aitken [39] and Rayleigh [40,41], respectively. Then, in 1994, François [42] first reported the honeycomb film formed by the breath figure method using star polystyrene (PS) polymer or PS/polyparaphenylene (PPP) block copolymer as building units (Figure 1). In this process, organic carbon disulphide (CS_2_) was used as the polymer solvent. François proposed three significant factors for honeycomb film formation: (1) material structure, (2) solvent, and (3) moist atmosphere. Since then, more and more materials and solvents have been found to be able to form honeycomb films. The mechanism was described as the sequence of a gelation process:

(a) Phase separation with rapid solvent evaporation;
(b) Polymer concentration increasing;
(c) Solution surface cooling;
(d) Water condensation.

**Figure 2:**
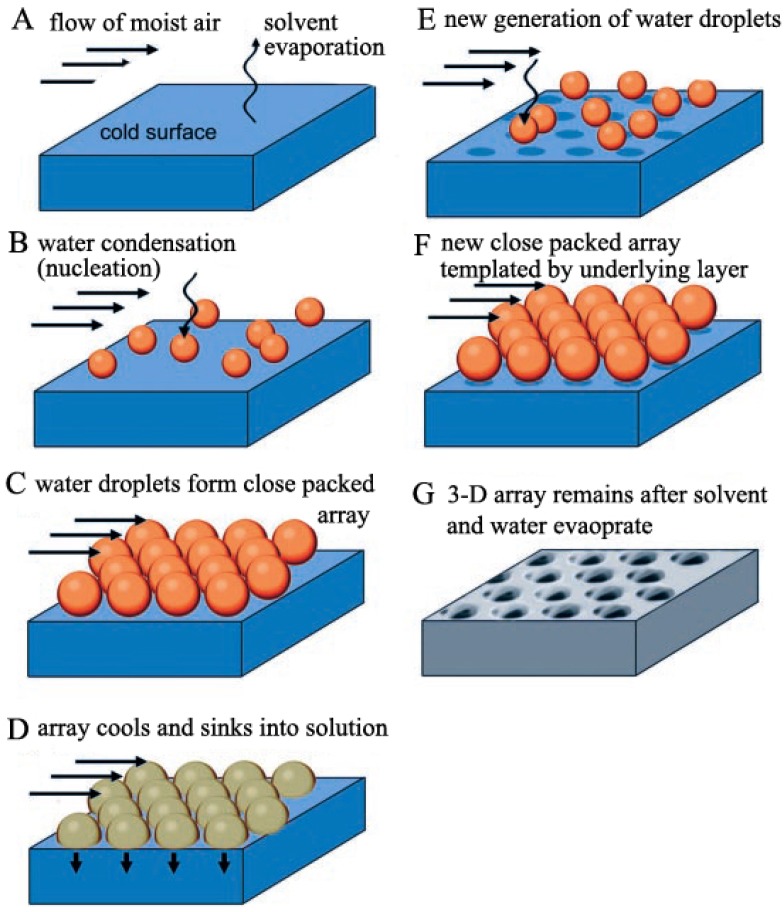
Illustration of the ordered honeycomb structure formation mechanism proposed by Srinivasarao. (**A**) solvent evaporation leads to the cold polymer solution surface; (**B**) water condenses on cold surface to become water droplets, which is the nucleation process; (**C**) water droplets from close packed array because of the convective currents of the evaporation and moist air flow; (**D**) water droplet array cools and sinks into solution; (**E**) new generation of water droplets; (**F**) new close packed array template by underlying layer; (**G**) 3-D array remains after solvent and water evaporation, which means the ordered honeycomb pores formation. The copyright permission was from reference [46].

Later, François [43,44] did more work on the mechanism exploration. Light-scattering experiments were carried out to observe the water droplets forming and growing on the surface of the polymer solution [43]. Additionally, no coalescence was observed during this process. They proposed that polymer precipitation at the solution/water interface was the decisive process. The polymer precipitated at the interface and then created a solid polymer envelope around the water droplets to avoid the water droplet coalescence [45]. Generally, the specific material (such as star PS), the organic solvent, and the flow of moist air were the key parameters for the breath figure process. To describe the mechanism more clearly, Srinivasarao [46] proposed the honeycomb film formation process as the following (Figure 2):

(a) Liquid surface becomes cold with the solvent evaporation;
(b) Water condenses on the cold surface to become water droplets, which is the nucleation process;
(c) Water droplets form a close packed hexagonal array because of the convective currents of the evaporation and moist air flow;
(d) Water droplet array cools and sinks into the polymer solution;
(e) New nucleation and growth of the moist air induce the formation of another water droplet layer;
(f) New layers of close packed hexagonal water droplet arrays are generated;
(g) The solution surface cools back to room temperature after the solvent is totally evaporated, along with the honeycomb film formation after water evaporation;
(h) Repeating the process from (d) to (f), another water droplet array layer forms.

Srinivasarao figured out that the water droplets could sink into the polymer solution, forming multilayer honeycomb films when the density of the solution was smaller than water. Otherwise, monolayer honeycomb films could be obtained if the water droplets could not sink into the solution.

**Figure 3:**
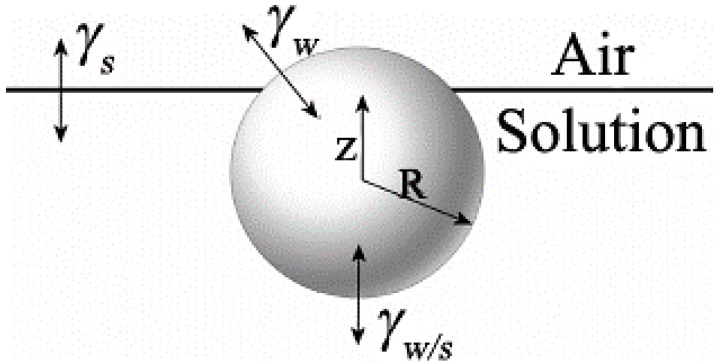
Schematic view of a spherical water droplet at the air/solution interface, with copyright permission from [47]. γ*_s_* and γ*_w_*, the surface tension of solution and water, respectively; γ*_w/s_*, the interfacial tension between water and the solution; *z*, the distance between the water droplet center and surface; *R*, the radius of the spherical water droplet.

To explain the mechanism of monolayer or multilayer formation, researchers have also proposed other mechanisms. Bolognesi and co-workers [47] explained that the interfacial energy between the water droplets and the organic solvent was the main factor determining the number of layers, as shown in Figure 3. The interfacial energy balance *z*_0_ is defined as:

*z*_0_ = *z*/*R* = (γ_w_ − γ_w_/_s_)/*γ*_s_ (1)

where *z* is the distance between the droplet center and the air/solution interface; *R* is the droplet radius; *γ*_w/s_ is the interfacial tension between water and solution; *γ*_w_ and *γ*_s_ are the surface tension of the water and the solution, respectively. When −1 < *z*_0_ < 1, one layer of droplets stayed between the air and solution interface, forming monolayer ordered structures. When *z*_0_ > 1, the droplets immerged into the solution, forming multilayer films. When *z*_0_ < −1, water droplets could not remain at the interface or in the solution, so no ordered structure could be obtained. Bolognesi [47] provided three examples as shown in Table 1. When CS_2_ was used as solvent, *z*_0_ < 1, a monolayer honeycomb film was formed, while the solvent of toluene and benzene induced the formation of multilayer films with *z*_0_ > 1.

**Table 1:** The honeycomb film structures predicted from *z*_0_ values, with copyright permission from [47].

Solvent	*γ* (mN/m) *T* = 20 °C	*γ*_H2O/solvent_ (mN/m) *T* = 20 °C	*z*_0_	Type of Structure
CS_2_	32.3	47.3	0.84	monolayer
toluene	27.9	36.1	1.30	multilayer
benzene	28.2	35	1.33	multilayer

**Figure 4:**
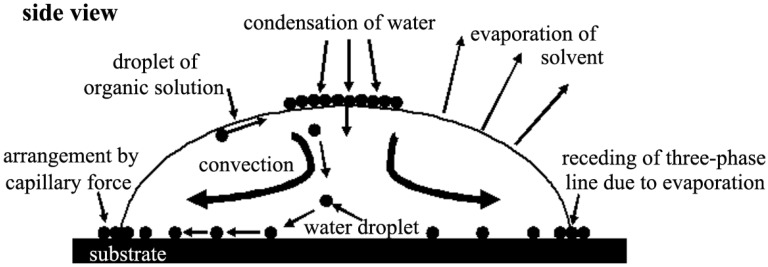
Top view of the honeycomb structure formation mechanism of polyion complexes, with copyright permission from [51].

During the formation mechanism study, driving forces attracted a lot of attention due to their important role in the film formation process [35,48,49,50]. Bormashenko *et al.* [48] added specific additives into polymer solution to promote the large-scale patterned films, resulting from the temperature gradient-induced Marangoni instability, where the Marangoni number was explained by an equation. Chin *et al.* [49] formed non-close-packed patterns by adding a heat source to provide the moderate temperature to generate the Marangoni force. Infrared (IR) thermographs showed that the droplet center was cooler than the droplet circumference after casting the polymer solution onto a glass substrate. According to the Marangoni effect [50], the colder center possessed higher surface tension, leading the fluid to move radially inward to the droplet center, which was in agreement with the observation that honeycomb patterns concentrated at the center of the circular films. Meanwhile, Maruyama [51] proposed a mechanism as shown in Figure 4. They believed that the Marangoni convection and capillary forces were the main driving forces [34,35,36,52,53,54,55]. The polyion complex was used as the building unit with chloroform as the solvent. Water droplets could align rather than coalesce because the polyion complex was surface-active. This was a crucial theory to instruct the process selection of building units to form honeycomb films. The convection force was the driving force for the water droplets sinking, and the capillary force was the driving force for the droplets arranging into an ordered array. This mechanism was different from the explanation of Srinivasarao, which has later been widely used by other researchers in their work.

**Figure 5:**
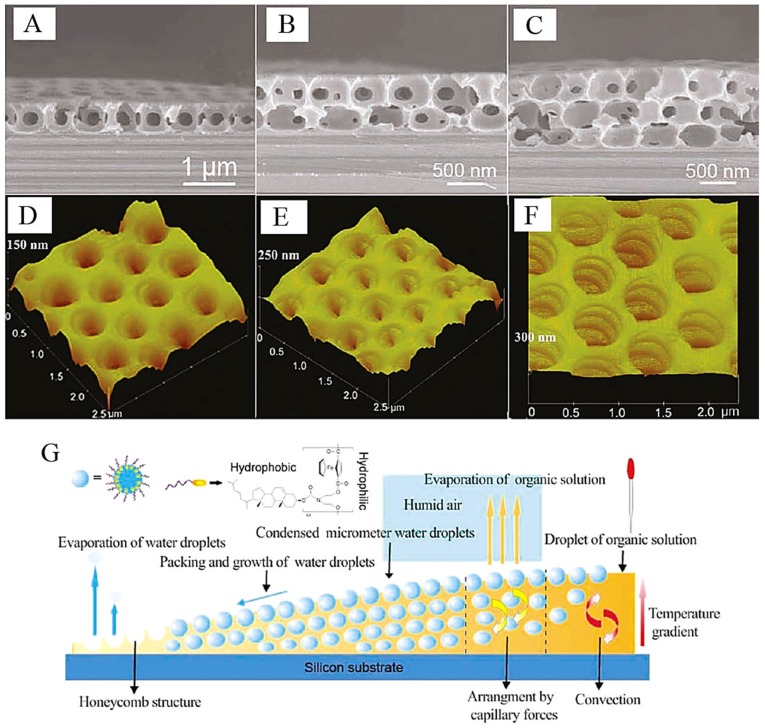
SEM (**A**–**C**) cross-section images and AFM (**D**–**F**) 3D observations of the monolayer (**A**,**D**), bilayer (**B**,**E**), and trilayer (**C**,**F**) honeycomb films from 0.6 mg/mL oligomer **1**/CS_2_ mixed solution on silicon substrates. (**G**) The schematic drawing of the formation mechanism of honeycomb structures from single-layer to multilayer arrays of the pores on a silicon substrate with ferrocenyl-cholesterol as the building unit, with copyright permission from [56].

Hao *et al.* also explored the mechanism of the honeycomb film formed by the breath figure method. For example, the amphiphilic ferrocenyl-based oligomer with cholesterol as side chains was used as the building unit to form honeycomb film on the solid silicon substrate and the air/water interface [56,57]. They agreed with François for the basic mechanism of the breath figure method and Maruyama for the explanation of driving forces for droplet formation and orderly packing. More importantly, they found that the formation of the monolayer or multilayer of honeycomb films depended not only on the organic solvent properties but also on the deposition space of the water droplets. From the solution edge to the center, honeycomb films with monolayer, bilayer, and trilayer pores were formed and observed by SEM and AFM (Figure 5A–F) [56] in turn. A direct view model of the monolayer, bilayer, and trilayer honeycomb film formation and its mechanism is shown in Figure 5G. The Marangoni convection and thermos-capillary effects were the basic driving forces, while the resistance forces were the viscous force and the buoyancy force. When the total driving force was larger than the resistance force, the water droplets could be dragged into the polymer solution. However, the formation of monolayer or multilayer films was also related to the polymer solution thickness. This meant that at the edge of the polymer solution, only monolayer films could be formed since there was not enough deposition space for more layers of water droplets. For this process, the solvent evaporation speed should be slow to ensure enough deposition space (wet thickness) and enough time for water condensing, sinking, and aligning.

Furthermore, based on honeycomb films, pincushion films were obtained by peeling off the top layer of honeycomb films with simple adhesive tape [58,59,60,61,62,63]. Yabu *et al.* found that pincushion structures showed greater hydrophobic property than normal honeycomb films [58,59]. Hydrophobic honeycomb films and superhydrophobic pincushion films were obtained with fluorinated polymer solution. Additionally, the maximum contact angle of pincushion films was even 170° [58]. The Wenzel model [64] and Cassie model [65] were used to explain this phenomenon, which meant that the wettability was related to the surface roughness and the surface components, respectively.

### 2.2. Influence Factors of the Honeycomb Film Formation by the Breath Figure Method

The factors affecting the honeycomb film formation include the building units, solvents, substrates, temperature, humidity, air flow velocity, and so on. To know how these factors affect the process could help us to deeply understand the breath figure mechanism of the honeycomb film formation.

#### 2.2.1. Building Units

**Figure 6:**
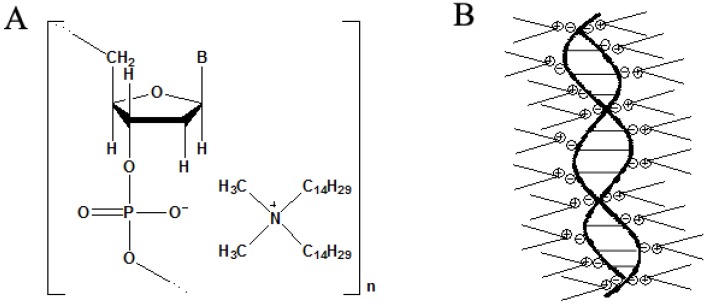
The chemical structure (**A**) and the schematic view (**B**) of the DNA-DTDA hybrid, with copyright permission from [69].

Since François found that star polymer and block copolymer could form honeycomb films in 1994, more and more materials have been explored in this area, including the hyper-branched polymers [52,54,66], the terminated linear polymers [35,47,52], and the normal linear polymers without a special terminal group [67,68], the amphiphilic polyion complexes [51], the organic-inorganic hybrids [64,65,66,67,68,69,70,71,72,73,74,75,76,77,78,79,80,81], and so on. Recently, many functional structures have been explored to construct honeycomb films with specific functions like bio-activity and fluorescent properties which could be applied in functional devices. For instance, the fluorescent property has been observed in the DNA/surfactant hybrid (Figure 6) honeycomb films with loaded RhB dye molecules. The surfactant used here is ditetradecyldimethylammonium (DTDA) [69].

As polymer was initially used to form honeycomb films, researchers have done a lot of work on the polymer honeycomb film formation process. The polymer structure, weight, and concentration all affect the honeycomb structures and properties. Polymer structures with special groups would endow the honeycomb films' functions [57,70,71,72,73,74,75,76,77]. For instance, the superhydrophobic honeycomb films were obtained with a fluorinated polymer [57,70], the thermo-responsive honeycomb films were due to the thermo-responsive amphiphilic copolymers [71], the photo-patterning honeycomb films were from polymers with groups sensitive to light (mainly UV light) [72,73,74,75,76], the electro-responsive films were achieved with liquid crystal polymers as building units [77].

According to Xu’s work, the honeycomb pore size decreased with polymer concentration increasing [78]. They used Henry's law to explain this phenomenon:

*P* = *P*_0_(1 − *X*_B_) (2)

where *P* and *P*_0_ refer to the vapor pressure of solvent in solution and the pure solvent, respectively;*X*_B_ is the mole fraction of the solute. Henry's law shows that the solvent of a solution with higher concentration owns lower vapor pressure, *i.e.*, a lower *P* value. Additionally, low vapor pressure slows the solvent evaporation, resulting in the higher surface temperature, which means a smaller temperature difference between the surface and atmosphere. Then the smaller temperature difference leads to slower droplet size increasing speed during the nucleation process. As the nucleation process is the key factor of determining the pore size, the higher solution concentration then means a smaller pore size.

Meanwhile, the polymer molecular weight remarkably affects the honeycomb pore size and depth, the distance between adjacent pores, and the specific porosity (defined as total pores area per unit area) [78,79,80]. For pore size, molecular weight is a positive effect. Xu *et al.* still explained it using Perry's law. The polymer mole fraction reduced with a higher molecular weight, leading to a higher *P* value and larger pore size. For pore depth, Matsuyama obtained a negative relationship, which meant that the pore depth decreased with the molecular weight increasing [79]. However, Gendelman *et al.* got the opposite relationship [80]. Gendelman deduced that this discrepancy was related to the peculiarities of the honeycomb pattern formation process in Gendelman’s and Matsuyama’s experiments.

Organic-inorganic hybrids have been regarded as promising honeycomb film-building units, in which the inorganic property and size and the organic chain length and surfactant type play important roles in the honeycomb film formation. The details will be discussed in next sessions.

#### 2.2.2. Solvents

In the honeycomb film formation process, the solvent evaporation speed needs to be high regardless of its relative density or miscibility to water. Many organic solvents have been found to be able to form honeycomb films with building units, such as carbon disulfide (CS_2_), chloroform (CHCl_3_), benzene, toluene, tetrahydrofuran (THF), and mixtures like CHCl_3_/CH_3_OH and CS_2_/toluene [57].

In 2004, Kim *et al.* found that multilayer honeycomb films were formed using water-immiscible solvents, such as CHCl_3_, while monolayer films formed using water-miscible solvents, such as THF with cellulose acetate butyrate (CAB) as the building unit [81]. However, in 2006, Kim *et al.* obtained the transition from monolayer to bilayer honeycomb films still using the CAB/THF solution but with decreasing polymer concentration, which was explained by the solvent evaporation speed and the interfacial energy between water droplets and the solvent [82]. Ferrari *et al.* [83] used liner PS to form honeycomb films by choosing proper solvents and substrates. The properties of solvents such as the thermodynamic affinity between the polymer and solvent, water miscibility, boiling point, and boiling enthalpy have also been found to be important for the honeycomb structure formation.

#### 2.2.3. Substrates

**Figure 7:**
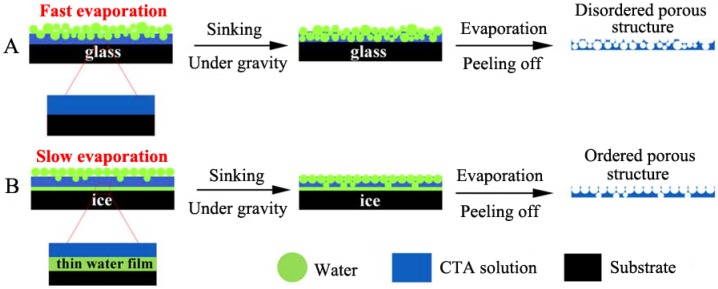
Comparison of the porous structure formation on glass (**A**) and ice (**B**) substrates, with copyright permission from [88].

Honeycomb films were formed on the surfaces of solid substrates at the beginning, and then the air/water interface from which the honeycomb film could be easily lifted off and transferred was also explored. The typical surface substrates for honeycomb film formation include mica [84], silicon [84,85], silicon oil surface [86,87], and ice [88]. Cong *et al.* [88] have compared the two substrates of glass and ice for the formation of the honeycomb film of cellulose triacetate (CTA) (Figure 7). It showed that the ice surface, on which ordered porous structures have been obtained, had a lower temperature and a lower water evaporation speed.

Ferrari *et al.* [83] tested honeycomb film formation on various inorganic and organic substrates, such as glass, silicon, silanized glass, fluorinated glass, polyethylene (PE), polyvinylchloride (PVC), polyethylene terephthalate (PET), *etc.* The orthogonal experimental data showed that there was an interaction between the substrate material and the solvent material, such as the wetting ability of the solvent on the substrate surface. As a result, for the CS_2_ solvent, the inorganic glass substrate and the organic PVC substrate were proper for honeycomb structure formation; for the dichloromethane solvent, glass, silanized glass (inorganic substrates) or PET, PVCs (organic substrates) were more suitable for honeycomb film formation.

#### 2.2.4. Temperature

**Figure 8:**
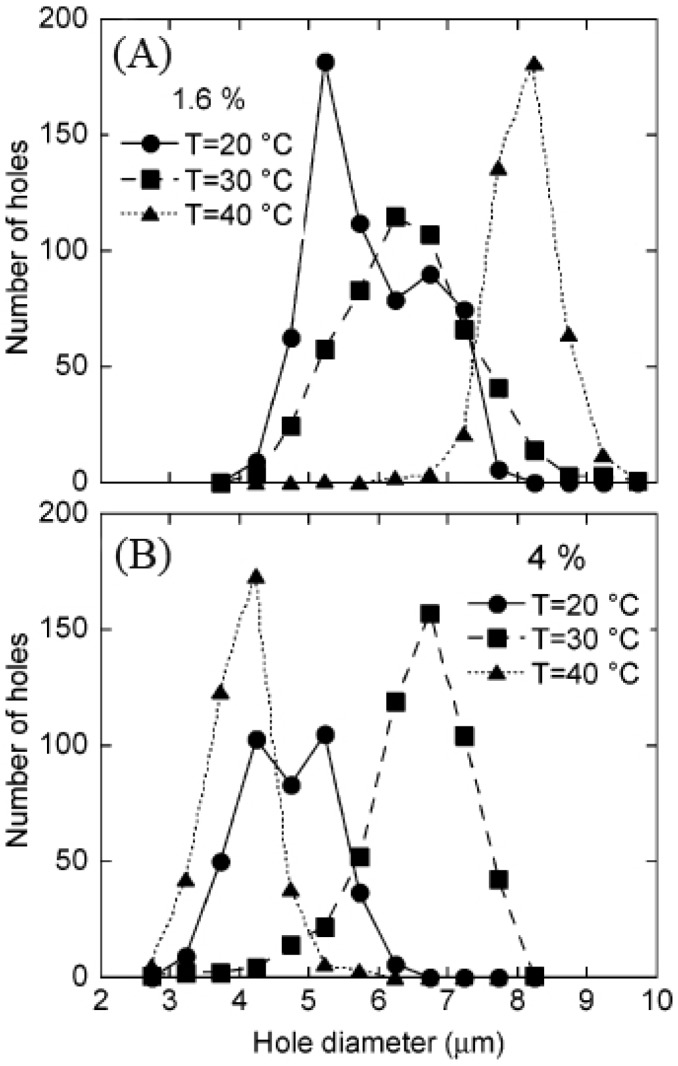
The pore size distribution of the PS100K-2COOH honeycomb films from CS_2_ solutions at different temperatures for samples with the polymer concentration of 1.6% (**A**) and 4% (**B**), with copyright permission from [47].

Honeycomb structure formation is sensitive to temperature [47,89]. Many experiments have confirmed that honeycomb structures can be obtained in a certain temperature range, and the temperature affects the pore size, pore size distribution, and pore shapes. Bolognesi *et al.* [47] prepared ordered honeycomb films with hydrophilic terminated linear polymer PS100K-2COOH dissolved in CS_2_ with the concentrations of 1.6% and 4% at temperatures of 20 °C, 30 °C, and 40 °C. As shown in Figure 8, when the temperature increased from 20 to 40 °C, for the 1.6% concentration sample, the pore size increased from 5 to 8 μm and the pore size distribution became narrower, while for the 4% concentration sample, the pore size was not directly related to the temperature changing, though the pore size distribution became narrow and the film changed from monolayer to multilayer.

Cong *et al.* [88] compared the difference between normal glass substrate and ice substrate, which was actually related to the substrate temperature. For the influence of substrate temperature [90,91,92], Chin *et al.* [90] observed non-close-packed (NCP) honeycomb arrays at higher substrate temperatures caused by the patterned metal stage beneath, and close-packed (CP) honeycomb pores of PS/(CHCl_3_/C_2_H_5_OH) organic solution. Meanwhile, Perry *et al.* [92] compared the temperature drop of the wafer edge and center with various solvents on glass or silicon substrates. The temperature drop was caused by solvent evaporation, and the temperature drop difference was due to the metal replenishing process of the wafer center. This temperature drop difference resulted in the evaporation difference and surface tension gradient, which caused the Marangoni effect and affected the film thickness in the end.

#### 2.2.5. Humidity

**Figure 9:**
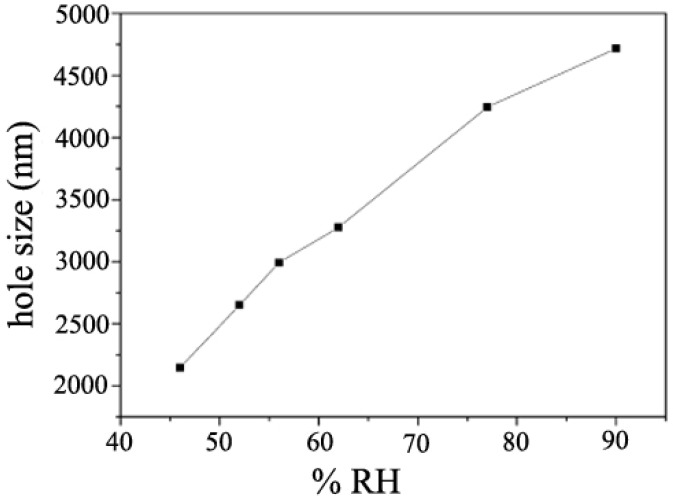
Curve of the pore size of PS honeycomb films *versus* the relative humidity, with copyright permission from [52].

The process humidity also plays a significant role in the honeycomb structure formation. On solid substrates, the honeycomb film formation process description typically needs to figure out the relative humidity data, while at the air/water interface, the humidity is normally enough [52]. Peng *et al.* [52] used PS (M_w_ = 223.2 k, 1 wt%) toluene solution to form honeycomb films, and found that the pore size of prepared PS honeycomb films increased with the relative humidity, as shown in Figure 9. During this process, the relative humidity was adjusted by the nitrogen flow speed, *i.e.*, the relative humidity increased from 40% to 95% with the nitrogen speed varying from 0 to 1 L/min. At low humidity of <46% or high humidity of >90%, no ordered structure formed because of the lack of water or too many droplets condensing onto the solution surface coalesced with adjacent water droplets.

#### 2.2.6. Other Factors

**Figure 10:**
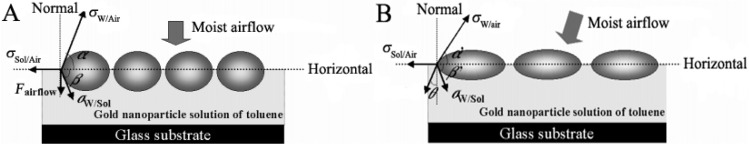
Models of water droplets as templates to form circular pores (**A**) or elliptic pores (**B**), with copyright permission from [93].

Air flow velocity has been found to affect the honeycomb film pore shape and size [93,94]. Normally, with higher air flow speed, smaller pores were formed. Li *et al.* [93] found that honeycomb structures with different aspect ratios of the elliptic pores were formed with a 15° direction of moist air flow at a different air flow velocity, while circular pores were formed with perpendicular air flow (Figure 10).

**Figure 11:**
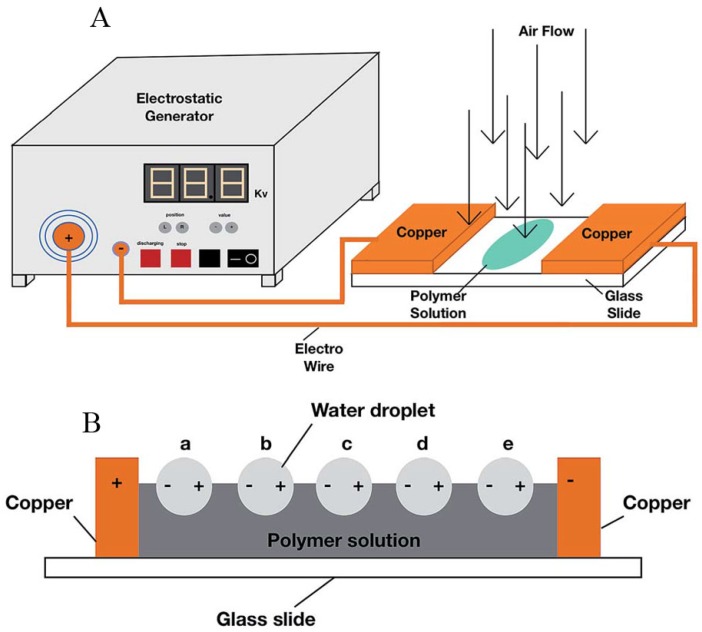

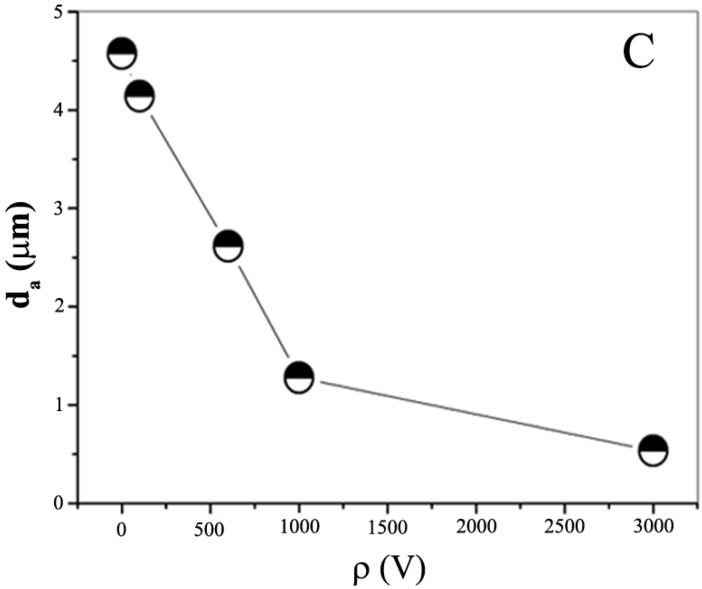
(**A**) Illustration of the electric breath figure device; (**B**) Schematic view of the electrostatic distribution of water droplets in the electric breath figure process, with copyright permission from [95]; (**C**) Curve of the pore diameter *versus* the applied voltage of PS/SiO_2_ chloroform solution, with copyright permission from [96].

The applied voltage has also been found to affect the formation of honeycomb films. Zhal *et al.* [95,96] applied voltage during the honeycomb film formation process by linking two electrodes to the two copper plates on glass surface, as shown in Figure 11A. This process was called the electric breath figure (EBF) process. The mechanism is explained in Figure 11B. The voltage created the electrostatic interaction between two adjacent water droplets. The water surface tension was found to decrease with increasing the applied voltage, leading to the decrease of the contact angle between the water droplets and the PS chloroform solution. When SiO_2_ nanoparticles were added to the PS solution to assist this process, the PS/SiO_2_ chloroform solution was casted on the glass surface between the two copper plates [96]. The PS/SiO_2_ hybrid honeycomb pore size decreased with increasing the applied voltage (Figure 11C). Furthermore, the honeycomb film became more hydrophobic with the increasing voltage. As a consequence, superhydrophobic film with a contact angle of 150.1° was obtained when the applied voltage was increased to 3000 V.

Chin’s group [49] prepared non-close-packed (NCP) honeycomb pore arrays of PS/CHCl_3_ solution using electrostatic interaction by applied voltage, which was different from Zhal’s work. PS was acidified with hydrogen bromide (HBr). Water droplets received H^+^ ions from HBr in polymer solution to obtain positively charged water droplets, resulting in the inter-droplet electrostatic repulsion, which was the main driving force for the droplets aligning into ordered arrays.

## 3. Organic-Inorganic Hybrids as Building Units to Form Honeycomb Films

**Figure 12:**
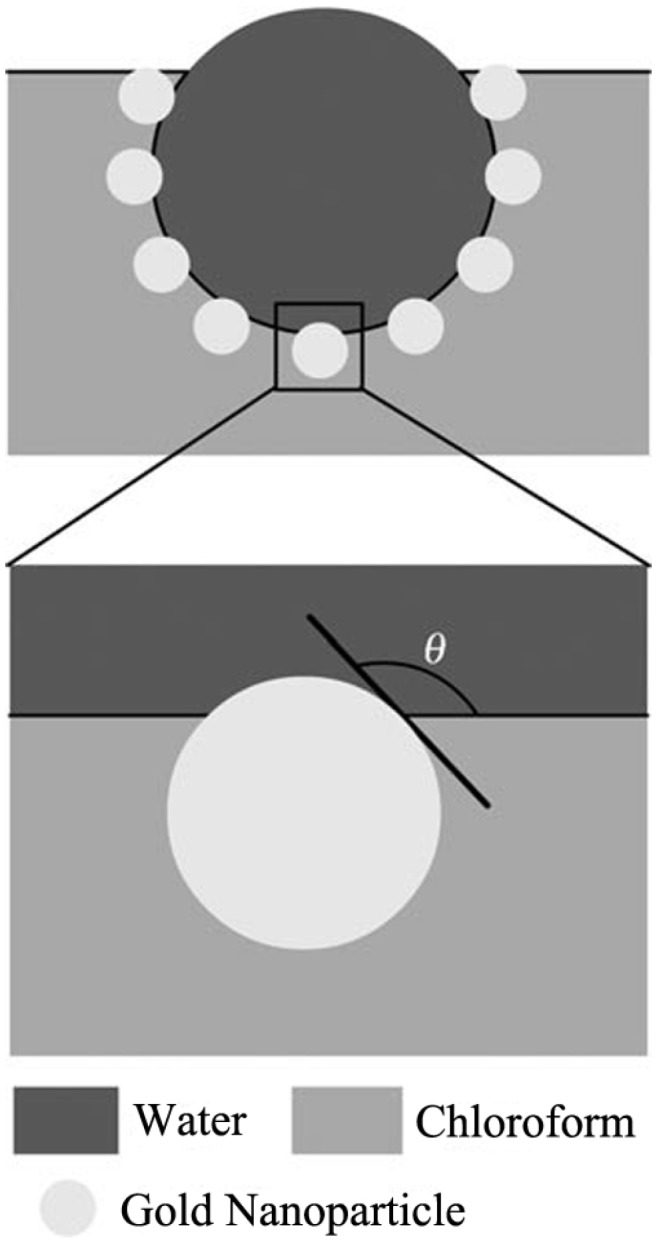
Schematic diagram of a water droplet stabilized by the dodecanethiol-stabilized gold nanoparticles at the water/chloroform interface, with copyright permission from [98]. Here, *θ* is the contact angle of a particle at the water/chloroform interface.

The organic-inorganic hybrid is a type of building unit used to form honeycomb films. The combination of the organic groups and inorganic groups builds the basic structure of honeycomb films, which also brings special properties, such as the surface-enhanced Raman effect, the surface hydrophobicity, and the fluorescent, magnetic, and photoelectric properties, *etc.* The organic part could be functional organic groups, such as surfactants or some bio-active organic groups. The inorganic part could be metal nanoparticles, POMs, QDs, and carbon nanotubes [97]. Water droplets are normally stabilized by these inorganic nanostructures modified by organic groups. A typical example is shown in Figure 12 [98]. The dodecanethiol-stabilized gold nanoparticles sit at the interface of a water droplet and the CHCl_3_ solution with a contact angle of θ, which is larger than 90° here, meaning that this nanoparticle has better wettability to CHCl_3_ solution than to water.

### 3.1. Surfactant Modified Nanoparticles

**Figure 13:**
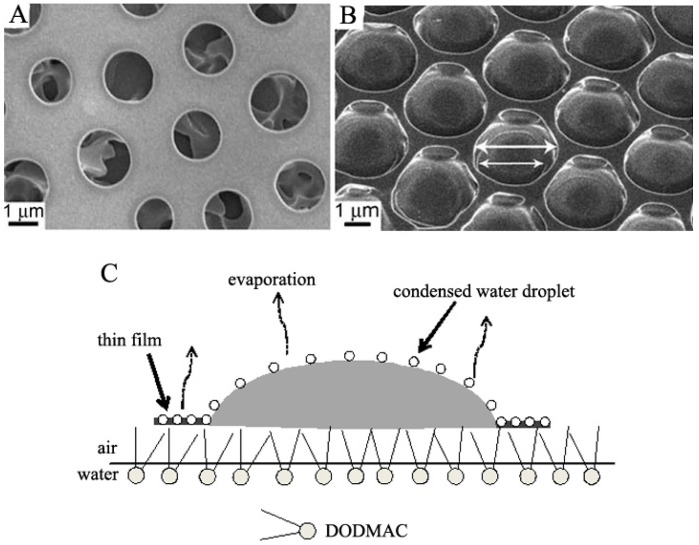
SEM images of the honeycomb structures formed at the gold nanoparticle concentration of 0.8 g/L (**A**) and 0.6 g/L (**B**); (**C**) Schematic diagram of the honeycomb film formation mechanism at air/water interface. The copyright permission was from [99].

Nanoparticles own great optical, electrical, and magnetic properties according to the size effect. To prepare honeycomb films with these properties, nanoparticles modified by organic groups were used, such as Au [98,99], Ag [73,100,101], Al_2_O_3_ [102,103], SiO_2_, TiO_2_, and ZnO nanoparticles [103]. Hao *et al.* have investigated the honeycomb film formation and mechanism using surfactant-modified Au nanoparticles. For example, dodecanethiol-capped gold nanoparticles were used to fabricate 1.7–3.5 μm pore size honeycomb film at the air/water interface with the assistance of another surfactant, dioctadecyldimethylammonium chloride (DODMAC), as illustrated in Figure 13C [99]. Differentiated from the solid substrate using moist air flow to provide high humidity, the air/water surface provides enough humidity by itself. The authors have also found that different pore structures could be formed at different Au nanoparticle concentrations. The two-dimensional perforated monolayer dominated when the Au nanoparticle concentration was 0.6 g/L (Figure 13A), while three-dimensional alveoli-like porous films were mainly formed at 0.8 g/L concentration (Figure 13B). At the same time, these honeycomb films formed by modified Au nanoparticles possessed the property of surface-enhanced Raman spectroscopy (SERS) according to the nanoparticle size effect.

**Figure 14:**
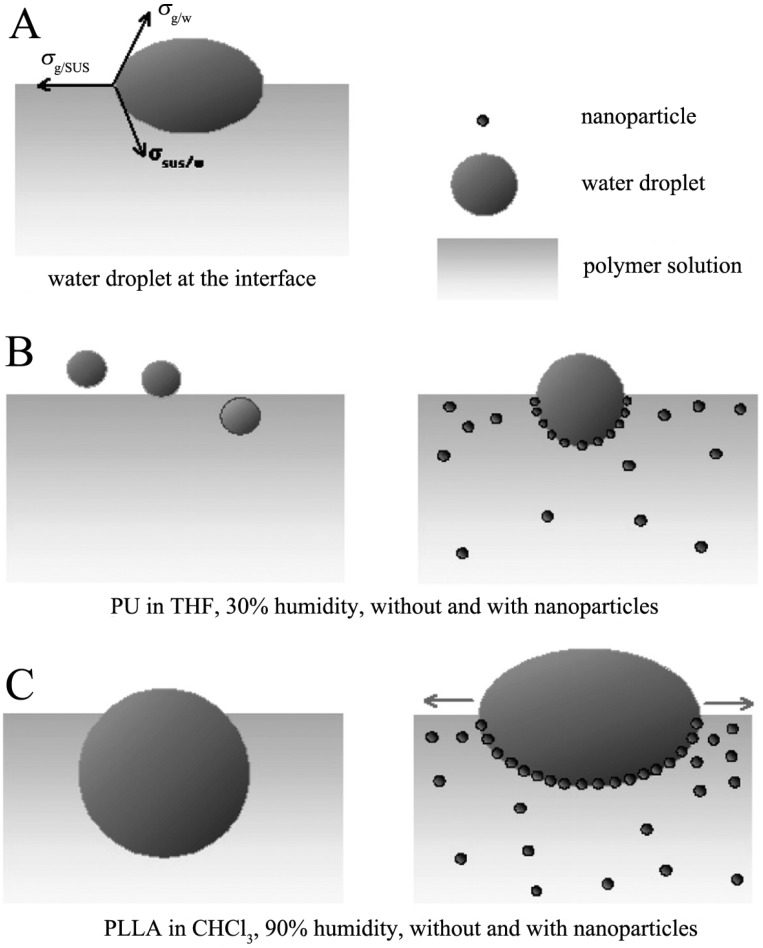
Schematic diagram of a water droplet at the air/solution interface with or without Ag nanoparticle stabilization during breath figure process. PU and PLLA are polyurethane and poly(L-lactic acid), respectively. (**A**) Wetting behavior of the water droplet at the solution-air interface. (**B**) PU in THF with 30% humidity, the water droplets sink into solution without the stabilization of Ag nanoparticles (left), while the water droplets spread stable at the solution-air interface with the stabilization of Ag nanoparticles (right). (**C**) PLLA in CHCl_3_ with 90% humidity, the water droplet grows fast to a large volume and immerses deeply into the polymer solution (left), resulting in the irregular pore pattern. And with the addition of Ag nanoparticles, the water droplet spreads at the solution-air interface stably with more surface exposed to air (right). The copyright permission was from [100].

Ag nanoparticles have also been found to be potential building units for constructing honeycomb films [100,101] because the aggregation of Ag nanoparticles can induce the breath figure process. Jiang *et al.* [100] found that the presence of Ag nanoparticles in polymer solution assisted the water droplets to spread on the air/polymer solution interface, as shown in Figure 14. Water droplets spread over a larger area on the interface with more surfaces into the air and less into the polymer solution.

### 3.2. Surfactant Modified POMs

**Figure 15:**
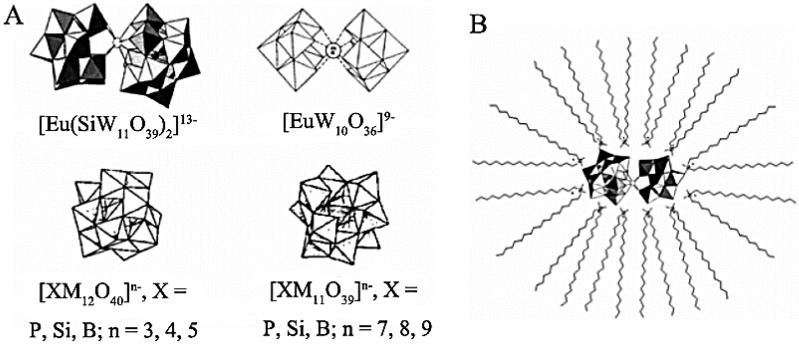
Schematic drawings of the structures of SECs series (**A**) and the SEC-1 structure (**B**), with copyright permission from [107].

Polyoxometalates (POMs) own great physical, chemical, and biological properties, showing potential applications in catalysis, energy storage, biomedicine, and other areas [104,105,106]. To expand their special properties in more fields, scientists created various honeycomb films using surfactant-modified POMs [105,106,107,108,109,110,111,112,113]. For example, Wu *et al.* prepared a series of supramolecular complexes, *i.e.*, different types of SECs (surfactant-encapsulated polyoxometalate complexes) with dimethyl dialkylammonium surfactants (DODA, DTDA, DDDA, DHDA), as shown in Figure 15 [107]. Then various honeycomb films were prepared using these supramolecular complexes. By comparing the alkyl length (hydrophobic property) and SECs size, Wu *et al.* [38] concluded that both the wettability and the size of the SECs played a significant role in honeycomb structure formation. The proper hydrophobicity (>90° contact angle) and proper large size (11.86 nm^3^ volume size succeeded, while 8.70 nm^3^ or 6.82 nm^3^ volume size failed) were preferred for ordered structure formation. Meanwhile, (DODA)_4_H-[Eu(H_2_O)_2_SiW_11_O_39_] (SEC-1) was taken as an example to explore the honeycomb-ordered structure formation process.

Hao *et al.* found that other kinds of POMs modified by surfactants could also form ordered honeycomb films, such as {Mo_72_Fe_30_}–DODMA complexes [109], (DODMA)_10_{Mn_2_Bi_2_W_20_}, (DDDMA)_10_{Mn_2_Bi_2_W_20_}, (CTA)_10_{Mn_2_Bi_2_W_20_} [110], DODMA-{Mn_2_Bi_2_W_20_}, {Mo_154_}, {Mo_132_}, {Mo_368_}, and {PW_12_}, *etc.* [111]. These films possessed special properties; for instance, the ordered honeycomb film of DODMA^+^-encapsulated {Mn_2_Bi_2_W_20_} owned ferromagnetism, while the ordered honeycomb film of DODMA^+^-encapsulated {Mo_368_} had great electrochemical properties [111].

### 3.3. Modified QDs

**Figure 16:**
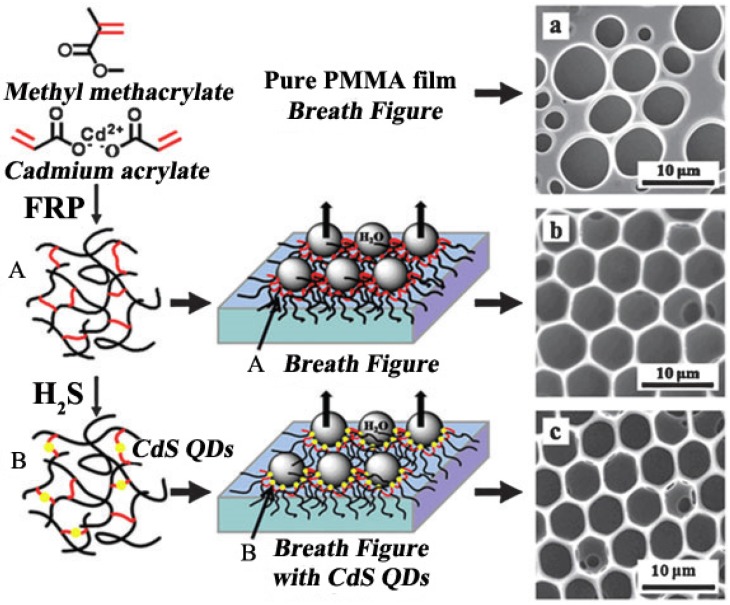
Illustration of the CdS QDs honeycomb film formation process. The initial process is the synthesis of poly(methyl methacrylate)/cadmium acrylate (PMMA/Cd(AA)_2_) ionomer, and the breath figure process of this ionomer is shown as (A); Then PMMA/CdS QDs was formed by exposing the ionomer to an H_2_S atmosphere, and the QD-polymer honeycomb film formation is shown as (B). The SEM images formed by these breath figure process are shown in (a) for pure PMMA film, (b) for PMMA/Cd(AA)_2_ ionomer film and (c) for PMMA/CdS QD-polymer film. Here, the solvent is CHCl_3_, polymer concentration = 6.7 wt%, Cd^2+^/S^2-^ = 1:0.33 mol mol^-1^, humidity = 85%. The copyright permission was from reference [114].

Chen *et al.* [114] exposed the PMMA/Cd(AA)_2_ ionomer of chloroform solution to an H_2_S atmosphere, obtaining ordered hexagonal-patterned PMMA/CdS films (Figure 16), which were polymer-QD hybrid honeycomb films, presenting an excellent fluorescence property.

Based on the breath figure method, polymer-QD hybrid (PS-CdTe) honeycomb films were obtained by dipping PS honeycomb film into aqueous CdTe nanocrystals. Galeotti [115] prepared the hybrid honeycomb films by combining the ordered hexagonal morphology feature and the CdTe QDs semiconducting photoluminescence feature, presenting potential applications in light-emitting devices and hybrid organic/inorganic solar cells. Böker [116] created the polymer-QD hybrid honeycomb films by the breath figure process of polymer solutions, along with the CdSe nanoparticle self-assembly process at the polymer solution and water interface. As a consequence, the ordered honeycomb pores were formed, and then the CdSe nanoparticle film was obtained along the polymer/air interface, forming tri-*N*-octylphosphine oxide (TOPO)-stabilized CdSe nanoparticle honeycomb films.

### 3.4. Applications of Organic-Inorganic Hybrid Honeycomb Films

**Figure 17:**
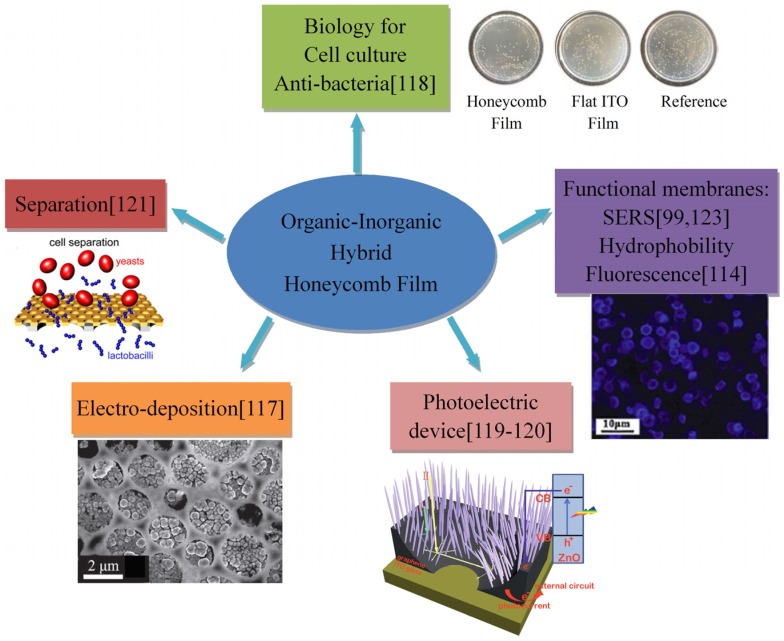
Summary of the applications of the organic-inorganic hybrid honeycomb films.

The honeycomb films formed by organic-inorganic hybrids possess the properties of both organic and inorganic parts, showing potential applications in many fields: electrochemistry [117], antibacterial treatment [118], photoelectric devices [119,120], separation [121], sensors [122], functional membranes (fluorescent, super-hydrophobic, SERS) [98,99,100,114,123], catalysis [99,100] and so on, as summarized in Figure 17. The honeycomb films formed by surfactant-modified Au or Ag nanoparticles could be used as the substrate of surface-enhanced Raman spectroscopy (SERS) [73,99,123]. POM-surfactant hybrid honeycomb films not only played an important role in biology, sensor, catalyst, and separation areas, but also showed the applicability for electro-deposition [106,107,108,109,110,111,112,113,117]. Hao *et al.* [117] developed film applications as electrode surfaces by transferring the film onto a solid surface after film formation. Then, Au nanoparticles were electrodeposited into the honeycomb macropores, obtaining a hierarchical structure.

## 4. Conclusions

Since François initially created ordered hexagonal honeycomb films by the breath figure method with star PS and block PS-PPP polymers in 1994, these films have attracted more and more attention. Currently, the research of breath figure honeycomb films has mainly been focused on exploring the mechanism, building unit expansion, factor adjustments, and application trials. In this review, we described the forming mechanism of the breath figure process. During the breath figure process, water droplets condensing into ordered arrays was the core part, and then the building units aggregated along the water droplets with further solvent and water evaporation to form ordered honeycomb films. As a consequence, the factors affecting the film formation were discussed, including the building units, solvents, substrates, temperature, humidity, air flow velocity, and the applied voltage. The organic-inorganic hybrid building units have been taken as an example to demonstrate the formation of honeycomb films with three types of materials: surfactant-modified nanoparticles, surfactant-modified POMs, and polymer-QDs. The organic-inorganic hybrid honeycomb films possess the properties of both organic and inorganic parts, showing potential applications in many areas.
